# The potential therapeutic role of extracellular vesicles in osteoarthritis

**DOI:** 10.3389/fbioe.2022.1022368

**Published:** 2022-09-16

**Authors:** Yu Zhuang, Shengjie Jiang, Changyong Yuan, Kaili Lin

**Affiliations:** ^1^ Department of Oral and Cranio-Maxillofacial Surgery, Shanghai Ninth People’s Hospital, Shanghai Jiao Tong University School of Medicine, Shanghai, China; ^2^ College of Stomatology, Shanghai Jiao Tong University, Shanghai, China; ^3^ National Center for Stomatology, National Clinical Research Center for Oral Diseases, Shanghai Key Laboratory of Stomatology, Research Unit of Oral and Maxillofacial Regenerative Medicine, Chinese Academy of Medical Sciences, Shanghai, China; ^4^ School of Stomatology, Xuzhou Medical University, Shanghai, China; ^5^ Department of Dental Implant, The Affiliated Stomatological Hospital of Xuzhou Medical University, Shanghai, China; ^6^ Shanghai Key Laboratory of Stomatology, Department of Oral and Cranio-Maxillofacial Surgery, Shanghai Ninth People’s Hospital, Shanghai Research Institute of Stomatology, Shanghai Jiao Tong University School of Medicine, Shanghai, China

**Keywords:** extracellular vesicles, osteoarthritis, mesenchymal stem cells, therapeutic treatment, EV engineering

## Abstract

Osteoarthritis (OA) is a worldwide and disabling disease, which cause severe pain and heavy socioeconomic burden. However, pharmacologic or surgical therapies cannot mitigate OA progression. Mesenchymal stem cells (MSCs) therapy has emerged as potential approach for OA treatment, while the immunogenicity and ethical audit of cell therapy are unavoidable. Compared with stem cell strategy, EVs induce less immunological rejection, and they are more stable for storage and *in vivo* application. MSC-EVs-based therapy possesses great potential in regulating inflammation and promoting cartilage matrix reconstruction in OA treatment. To enhance the therapeutic effect, delivery efficiency, tissue specificity and safety, EVs can be engineered via different modification strategies. Here, the application of MSC-EVs in OA treatment and the potential underlying mechanism were summarized. Moreover, EV modification strategies including indirect MSC modification and direct EV modification were reviewed.

## Introduction

Osteoarthritis (OA) is a worldwide and disabling disease, which cause severe pain and heavy socioeconomic burden (Glyn-Jones et al., 2015). The pathogenic risk factors for OA include trauma, aging, obesity, inheritance, etc. (Cisternas et al., 2016). OA is characterized as synovitis, degrading cartilage, damaged menisci and ligaments, and pathologically formed osteophytes (Loeser et al., 2012). Aging-related cell senescence, metabolic disorder and aberrant mechanical load can lead to senescence-associated secretory phenotype (SASP) release and local inflammation, which in turn aggravate cell senescence and cartilage matrix degradation (Glyn-Jones et al., 2015; Appleton, 2018; Boulestreau et al., 2021). Existing therapies for OA are symptomatic strategies like nonsteroidal anti-inflammatory drugs (NSAIDs), and surgical strategies like joint replacement (Tao et al., 2017). However, pharmacologic or surgical therapies cannot mitigate OA progression, or enhance damaged cartilage reconstruction. Recently, mesenchymal stem cells (MSCs) therapy has emerged as potential approach for OA treatment, while the immunogenicity and ethical audit of cell therapy are unavoidable (Zhuang et al., 2022). Effective strategies for inflammation regulation and cartilage regeneration are urgently required.

Extracellular vesicles (EVs), serving as cell-cell communication media, play a vital role in regulating tissue homeostasis and biological process. EVs derived from MSCs (MSCs-EVs) inherit the valuable characteristics of donor MSCs (Liu A. et al., 2021; Liu L. et al., 2021; Zhang X. et al., 2022). Compared with stem cell strategy, EVs induce less immunological rejection, and they are more stable for storage and *in vivo* application (Brennan et al., 2020). Multiple components (including miRNAs, lipids, proteins) make EVs potential candidates in promoting tissue regeneration and modulating immunity (Yu et al., 2022). MSCs-EVs have been reported to improve chondrocyte phenotype, attenuate cartilage degradation *in vitro* and ameliorate OA progression *in vivo* (Zhang S. et al., 2016; Tofiño-Vian et al., 2018; Woo et al., 2020). To enhance the therapeutic effect, delivery efficiency, tissue specificity and safety, EVs can be engineered via different modification strategies.

**FIGURE 1:**
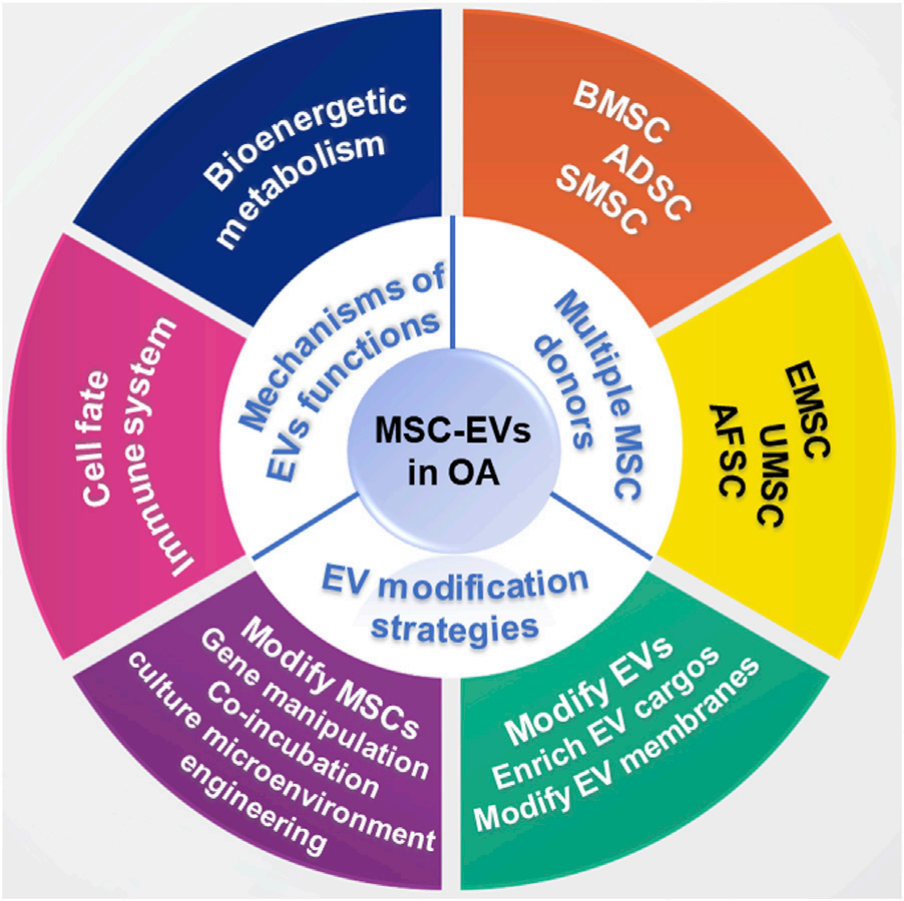
Schematic illustration for the application of MSC-EVs in OA treatment.

EV-based therapy possesses great potential in regulating inflammation and promoting cartilage matrix reconstruction in OA treatment. In this review, we summarize the application of MSC-EVs in OA treatment and the potential underlying mechanism. Moreover, EV modification strategies including indirect MSC modification and direct EV modification were also reviewed (Figure 1).

## The application of extracellular vesicles in osteoarthritis treatment

There have been more and more studies indicating that MSC-EVs are potential in controlling inflammation, inhibiting cartilage matrix degradation, and promoting cartilage repair for OA treatment. The applications of EVs derived from different MSCs in OA treatment and the potential underlying mechanisms are summarized here.

### The therapeutic role of different MSC-derived extracellular vesicles

The cargos of EVs may vary depending on their donor cells and consist of multiple bioactive molecules including proteins, lipids, and miRNAs, thus leading to specific characteristics of different MSC-EVs (Mianehsaz et al., 2019). MSCs therapy has been proved to attenuate inflammation, prevent cartilage matrix degradation, and ameliorate pain in clinical trials (Toh et al., 2017; Song et al., 2020). The mechanism underlying the MSCs therapeutic effect might be secretion of bioactive molecules (Eleuteri and Fierabracci, 2019), additionally, application of EVs secreted from MSCs possess inherent advantages compared to direct MSC therapy (lower immunogenicity, tumorigenicity, etc.) (Ankrum et al., 2014; Fichtel et al., 2022). Thus, EVs therapy has gained more and more attention in disease treatment and tissue reconstruction. EVs derived from different MSCs source for OA treatment have been summarized here (Table 1).

**TABLE 1:** The therapeutic role of different MSC-EVs in OA treatment.

MSC source	Cargo	Model	Delivery strategies	Therapeutic effect	References
BMSCs	miR-92a-3p	Collagenase induced mice OA model	Local intra-articular injection	Promoting cartilage development and maintaining homeostasis via miR-92a-3p/pathway	Mao et al. (2018)
	miR-320c	Chondrocytes isolated from OA articular cartilage samples	Co-culture	Enhancing cartilage extracellular matrix deposition (upregulate SOX9 and downregulate MMP13)	Sun et al. (2019)
	—	Collagenase induced mice OA model	Local intra-articular injection	Inhibiting inflammation, inducing expression of matrix formation-related genes and preventing OA progression	Cosenza et al. (2017)
	lncRNA MEG-3	Anterior cruciate ligament (ACL) transection and medial meniscectomy (MM) induced rat OA model	Local intra-articular injection	Reducing the senescence and apoptosis of chondrocytes	Jin et al. (2021)
	lncRNA LYRM4	IL-1β induced inflammatory chondrocyte	Co-culture	Reversing the carbolic changes of chondrocytes induced by IL-1β via lncRNA LYRM4-AS1/GRPR/miR-6515–5p pathway	Wang et al. (2021a)
	miR-136–5p	Post-traumatic mice OA model	Local intra-articular injection	Promoting collagen II, aggrecan, and SOX9 expression of chondrocytes via miR-136–5p/ELF3, and inhibiting post-traumatic OA progression	Chen et al. (2020)
	—	Anterior cruciate ligament (ACL) transection induced rat OA model	Local intra-articular injection	Alleviating OA via promoting M2 polarization of synovial macrophages	Zhang et al. (2020)
	lncRNA NEAT1	Destabilization of the medial meniscus (DMM) induced mice OA model	Local intra-articular injection	Activating the proliferation and autophagy of chondrocytes via lncRNA NEAT1/miR-122–5p/Sesn2/Nrf2 pathway	Zhang and Jin, (2022)
ADSCs	—	IL-1β induced inflammatory chondrocyte	Co-culture	Inhibiting inflammation and protecting chondrocytes via upregulating annexin A1 and downregulating NF-κB	Tofiño-Vian et al. (2018)
	miR-199a, 125b, 221, 92a)	Destabilisation of the medial meniscus (DMM) induced mice OA model	Local intra-articular injection	Enhancing cartilage matrix deposition and protecting cartilage from degradation	Woo et al. (2020)
	—	IL-1β induced inflammatory chondrocyte	Co-culture	Attenuating inflammatory micro-environment via inhibiting NF-κB pathway	Cavallo et al. (2021)
EMSCs	—	MIA injection induced rat TMJ-OA	Local intra-articular injection	Activating cartilage repair and restoring matrix via activating adenosine receptor, and phosphorylation of AKT, ERK and AMPK	Zhang et al. (2019)
UMSCs	miR-100–5p	Chondrocytes isolated from OA articular cartilage samples	Co-culture	Inhibiting ROS production and cell apoptosis through miR-100–5p/NOX4	Li et al. (2021d)
	miR-122–5p, 148a-3p, 486–5p, let-7a-5p, 100–5p	Anterior cruciate ligament (ACL) transection induced rat OA model	Local intra-articular injection	Enhancing M2 polarization through PI3K/AKT pathway, and alleviating OA progression	Li et al. (2022)
	miR-1208	Destabilisation of the medial meniscus (DMM) induced mice OA model	Local intra-articular injection	Reducing osteophyte production, and chondrocyte apoptosis via miR-1208/METTL3 induced m6A level decrease of NLRP3 mRNA	Zhou et al. (2022)
AFSCs	TGF-β	Monoiodoacetate-induced rat OA model	Local intra-articular injection	Modulating macrophage polarization and preventing cartilage damage	Zavatti et al. (2020)
SMSCs	miR-129–5p	IL-1β induced inflammatory chondrocyte	Co-culture	Suppressing IL-1β-mediated OA via miR-129–5p/HMGB1 pathway	Qiu et al. (2021)
	miR-26a-5p	IL-1β induced inflammatory chondrocyte	Co-culture	Inhibiting apoptosis and inflammation of chondrocytes	Lu et al. (2021)

EVs derived from bone marrow mesenchymal stem cells (BMSC-EVs) has been widely applied for OA treatment. BMSC-EVs can play a role in promoting the proliferation and matrix components secretion of chondrocytes, and improve the framework of subchondral bone. He et al. (2020) treated OA rats with BMSC-EVs, and the results indicated that BMSC-EVs improved chondrocyte phenotype and alleviated pain *via* ameliorating function of dorsal root ganglion (DRG). Moreover, BMSC-EVs were reported to regulate inflammation through restraining NF-κB pathway (Li et al., 2020), controlling inflammation related factor Autotaxin-YAP (Wang Y. et al., 2021), modulating macrophagocyte polarization (Zhang et al., 2020), and prevent chondrocyte apoptosis (Chen et al., 2020; Wang X. et al., 2021; Jin et al., 2021).

It has been reported that adipose mesenchymal stem cells (ADSCs) also showed potential in protecting cartilage (ter Huurne et al., 2012; Baharlou et al., 2017), and ADSC-EVs could play a role in regulating inflammation. (Mortati et al. (2020) utilized ADSC-EVs for OA treatment, and the results indicated that ADSC-EVs effectively promoted M2 polarization, inhibited inflammation and promoted cartilage matrix deposition. Apart from chondrocytes, ADSC-EVs could target synovial cells, modulating the synthetase, catabolic enzymes and inflammatory cytokines secretion of synovial cells, and positively improve the biological performance of EVs secreted by endogenous synovial cells and chondrocytes (Cavallo et al., 2021). Promoting autophagy of chondrocyte *via* mTOR pathway could also be one of the mechanisms under the therapeutic effect of ADSC-EVs in preventing OA process (Wu et al., 2019).

Human perinatal stem cells, with outstanding self-renewal capacity, are widely applied in OA treatment (Matas et al., 2019). More and more studies indicated that EVs derived from perinatal stem cells, maintaining the excellent traits of donor cells, are ideal alternatives to MSCs in cartilage repair (Tang et al., 2021; Zhang Q. et al., 2022; Zhang S. et al., 2022; Li et al., 2022; Zhou et al., 2022). EVs derived from embryonic MSCs (EMSCs) were proved to inhibit inflammation, reconstruct cartilage matrix, and alleviate pain in OA model (Zhang S. et al., 2016; Zhang et al., 2019). Umbilical cord MSCs (UMSCs) also secreted EVs that were capable to control inflammation *via* promoting M2 polarization and inhibiting m6A of NLRP3 in macrophages, and enhance cartilage repair (Park et al., 2017; Li X. et al., 2021; Zhang Q. et al., 2022; Li et al., 2022; Zhou et al., 2022). It has been reported that EVs derived from amniotic MSCs (AMSCs) (Silini et al., 2017; Ragni et al., 2021) and amniotic fluid stem cells (AFSCs) (Maraldi et al., 2013; Zavatti et al., 2020) are also potential in inflammation modulation and OA treatment.

In addition, synovial mesenchymal stem cells (SMSCs) showed stronger potential in chondrocyte differentiation. EVs derived from SMSCs showed great potential in immunomodulation and cartilage repair, and were applied in OA treatment (Zhu et al., 2017; Lu et al., 2021; Qiu et al., 2021).

### The potential mechanism under the therapeutic effect of MSC-derived extracellular vesicles

MSCEVs can effectively promote the synthesis of cartilage extracellular matrix (ECM) (Yeo et al., 2013). The mechanisms under the therapeutic effect of MSC-EVs attract much attention. Multiple MSCs can serve as donor cell source for EV production, and there are masses of various cargos in MSC-EVs, like nucleus acids and proteins. Considering that EVs derived from different MSCs possess similar therapeutic effect, MSC-EVs may share evolutionary conserved key bioactive molecules in their biological activity (Yeo et al., 2013; Toh et al., 2017). Proteins in MSC-EVs contain some housekeeping enzymes that play a role in reconstructing cartilage homeostasis *via* modulating cell fate of chondrocyte, remodeling bioenergetic metabolism, regulating immune system and promoting cartilage matrix synthesis (Lai et al., 2015).

#### Modulating cell fate of chondrocyte

In OA cartilage micro-environment, oxidative stress, ROS production and inflammatory factors boost intensively, which usually lead to cell apoptosis, cell death and cell dysfunction (Haslauer et al., 2013; Heard et al., 2015; Toh et al., 2016). There have been studies reporting that MSC-EVs could promote cell proliferation *via* activating the phosphorylation of ERK1/2 and AKT, the factors tightly connected with cell survival. In the repair process of damaged sites, excessive ATP can lead to cell death of neighbouring healthy cells. The ATP is metabolized *via* hydrolysis into AMP (Toh et al., 2017). CD73, the hallmark of EVs, serve as catalyst to activate the hydrolysis of AMP into adenosine, the activator of survival related enzymes (Colgan et al., 2006; Jacobson and Gao, 2006; Toh et al., 2017). The ability of MSC-EVs in converting ATP into pro-survival kinases make them potential in promoting cell proliferation for cartilage repair. Additionally, MSC-EVs are capable to inhibit apoptosis *via* mTOR pathway (Li X. et al., 2021; Jin et al., 2021; Lu et al., 2021; Zhou et al., 2022), and promote autophagy (Zhang and Jin, 2022) to improve cell performance of chondrocytes.

#### Remodeling bioenergetic metabolism

Mitochondria, the ATP production organelle, plays a key role in cartilage bioenergy homeostasis. Chondrocytes in OA are reported to suffer mitochondrial dysfunction and reduced electron transport chain (ETC) proteins activity. The inhibited ETC activity and ATP production in chondrocytes lead to abnormal bioenergetics, which then induce increased cell apoptosis, more ROS production, enhanced catabolism and inhibited anabolism of cartilage matrix (Vaamonde-García et al., 2012; Lee et al., 2020). MSC-EVs are enriched in enzymes to promote ATP production for decreased ATP generation compensation in defective chondrocytes, which make them potential in reconstructing bioenergetic homeostasis and repair capability of chondrocytes in OA (Pashoutan Sarvar et al., 2016; Toh et al., 2017).

#### Regulating immune system

Immune system is activated rapidly following tissue repair happening, which exerts vital influence on tissue reconstruction. Immune cell like macrophage, neutrophil and synovium cell release amount of pro-inflammation factors (IL-1β, IL-6, IL-8, MMPs, etc.), which then induce the cartilage matrix destruction and OA progression (Haslauer et al., 2013; Heard et al., 2015; Mianehsaz et al., 2019). Moreover, the modulation of macrophage M1-M2 polarization plays a role in maintaining inflammation balance during tissue repair process (Ding et al., 2016; Utomo et al., 2016). There have been many studies indicated that immunomodulation factors in MSC-EVs can synergistically reduce IL-1β, IL-6, TNF-α expression, promote M2 polarization, and enhance IL-10, TGF-β1 secretion, to construct a positive immuno-microenvironment for cartilage repair.

#### Promoting cartilage matrix synthesis

ECM is the important component of cartilage structure and gradually destroyed during OA progression. The inflammatory pathological micro-environment tends to induce cartilage matrix degradation, and cartilage structure loss (Haslauer et al., 2013; Heard et al., 2015). Activating reparative responses and anabolic related gene expression of chondrocytes is important in promoting cartilage matrix re-deposition (Mizuta et al., 2004). MSC-EVs are proved to promote cartilage matrix deposition *via* upregulating SOX9, aggrecan, col2 expression and inhibit matrix degradation through downregulating MMP13, MMP3, ADAMTS-5 expression (Cosenza et al., 2017; Sun et al., 2019; Zhang et al., 2019; Chen et al., 2020; Woo et al., 2020; Wang X. et al., 2021).

## The application of engineered extracellular vesicles in osteoarthritis treatment

MSC-EVs exert positive influence on immunomodulation, cell fate regulation, bioenergy homeostasis remodeling, and matrix synthesis modulation, which make MSC-EVs considered to be potential candidates for OA treatment. To further improve cargo delivery, cell specification and fusion efficiency, multiple strategies can be utilized to modify EVs, including direct (modify cargo and membrane of EVs) and indirect (modify MSCs) methods (Table 2).

**TABLE 2:** Modification strategies for EV engineering.

Modification methods		Approaches	Results	References
Indirect MSC modification strategies	Manipulating gene transfection	Virus transfection	Overexpressing miR-140–5p, and alleviating OA progress through downregulating VEGFA	Liu et al. (2022)
		Plasmid transfection	Upregulating circRNA_0001236, and inhibiting cartilage degradation via miR-3677–3p/Sox9	Mao et al. (2021)
		Plasmid transfection	Overexpressing lncRNA H19, and promoting chondrogenesis through miR-29b-3p/FOXO3	Yan et al. (2021)
	Co-incubating donor cells with bioactive molecules	Co-incubation with curcumin	Reducing the oxidative stress and protecting chondrocytes	Xu et al. (2022)
		Co-incubation with TGF-β1	Enhancing the M2 polarization via miR-135b/MAPK6 axis	Wang and Xu, (2021)
		Co-incubation with IL-1β	Inhibiting inflammation of OA	Kim et al. (2021)
		Co-incubation with LPS	Inhibiting cartilage matrix degradation	Duan et al. (2021)
	Engineering cell culture micro-environment	3D culture	Promoting chondrogenesis	Yan and Wu, (2020)
		Dynamic mechanical stimulation	Inhibiting inflammation via NF-κB signal pathway	Liao et al. (2021)
		Hypoxia micro-environment culture	Enhancing cartilage repair	Rong et al. (2021)
Direct EV modification strategies	Enriching EV cargos	Direct mixture method	Loading COS into EVs, and promoting anabolic related genes expression of chondrocytes	Li et al. (2021a)
		Electroporation	Loading KGN into EVs, and improving cartilage repair	Xu et al. (2021)
	Modifying EV membrane	E7 peptide modifying EV surface	Targeting synovial fluid-derived MSCs	Xu et al. (2021)
		Fusing CAP with EV surface protein	Improving the chondrocyte target ability	Liang et al. (2020)
		Modifying EVs with PPD	Regulating EV surface charge potential, and promoting EV penetration into cartilage matrix	Feng et al. (2021)

### Modifying MSCs for EVs engineering

EVs tend to inherit characteristics of their donor cells, and donor MSCs can be modified to obtain correspondingly customized miRNA enriched EVs (Kosaka et al., 2010; Ni et al., 2020). The advantage of indirect MSC modification lies in that the stimulation factors on MSCs are in control and can be quantified.

#### Manipulating gene transfection

Gene transfection manipulation, mainly *via* viral vectors, can not only improve EV yields, but also enhance functional cargos like miRNA, circRNA and LncRNA in engineered EVs for OA treatment Kosaka et al. (Kosaka et al., 2010) found that upregulating the expression of neutral sphingomyelinase 2 (nSMase2) in donor cells promoted the secretion of miRNAs, which are transferable and functional to target cells.

The functions of miRNAs are important in the biological performance of EVs in recipient cells. Through Microarray analysis and literature review, key miRNAs can be found out. Virus infection can effectively manipulate target gene expression of donor cells and cargos of EVS. It has been reported that upregulating expression of miR-26a–5p (Jin et al., 2020), miR-126–3p (Zhou et al., 2021), miR-155–5p (Wang Z. et al., 2021), miR-140–5p (Liu et al., 2022) in MSCs and EVs can effectively inhibit inflammation, matrix degradation, cell apoptosis and improve matrix secretion of chondrocytes. The miRNAs can bind to target mRNAs, induce mRNA degradation, and impact downstream signal pathways (Zhuang et al., 2022). Apart from miRNAs, circRNAs and lncRNAs are also potential in regulating cell behavior. CircRNAs like circRNA_0001236 (Mao et al., 2021), circHIPK3 (Li et al., 2021b) and lncRNAs like lncRNA H19 (Yan et al., 2021; Yang et al., 2021) can interact with target miRNAs, regulate downstream genes, inhibit catabolism and attenuate anabolism of chondrocytes. Thus, overexpression of functional circRNAs and lncRNAs in EVs can also be potential candidates for better OA therapeutic strategy.

#### Co-incubating donor cells with bioactive molecules

Bioactive molecules can be loaded into EVs *via* co-incubation with donor cells. The characteristic of donor cells obtained following compound co-incubation can be transmitted to EVs. Co-incubated with anti-inflammatory factors like curcumin (Li et al., 2021c; Xu et al., 2022), TGF-β1 (Wang and Xu, 2021) endows EVs with ideal capacity to modulate macrophage polarization, reduce oxidative stress and promote chondrocyte anabolism. Interestingly, IL-1β (Colombini et al., 2021; Kim et al., 2021) and LPS (Duan et al., 2021) preconditioned donor cell derived EVs are proved to inhibit inflammation, improve chondrocyte performance and ameliorate OA progression. The reason accounting for this lies in that MSCs treated with low concentration of LPS or IL-1β response adaptively, which is beneficial for immunomodulation.

#### Engineering cell culture microenvironment

**FIGURE 2:**
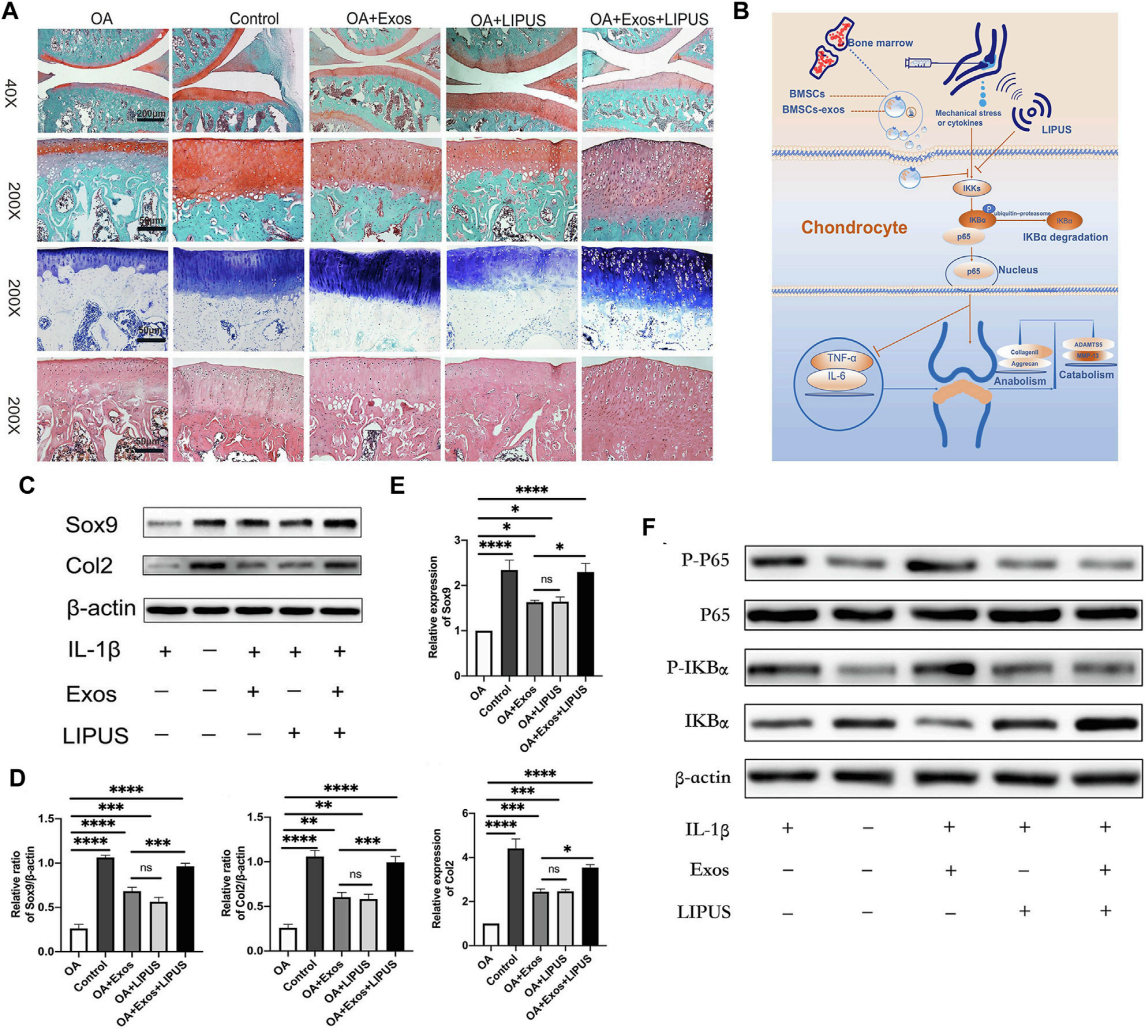
Low-intensity pulsed ultrasound (LIPUS) dynamic mechanical stimulus promoted the biological performance of MSC-EVs in OA treatment. **(A)** Safranin O, Toluidine Blue and HE staining for knee joints sections; **(B)** The mechanism underlying the therapeutic effect of LIPUS-treated MSC-EVs; **(C,D)** Western blot analysis for anabolism related proteins of chondrocytes, and semi-quantification; **(E)** RT-qPCR analysis for the expression of anabolism related genes; **(F)** Western blot analysis for the expression of NF-κB pathway related proteins. Reproduced from Ref. (Liao et al., 2021), International Immunopharmacology, ELSEVIER Publication at 2021.

MSCculture microenvironment changes can exert influence on the biological effect of EVs. Compared to 2D culture, 3D culture can enhance the yield and functions of EVs (Rocha et al., 2019). Yan et al. (Yan and Wu, 2020) constructed a hollow-fiber bioreactor to build a 3D culture microenvironment for cartilage restoration, and the results indicated that EVs isolated from 3D culture cells showed greater potential in promoting TGF-β1 expression and Smad2/3 pathway. Moreover, dynamic mechanical stimulation is also proved to be able to modulate cell proliferation and differentiation, and improve EV production and functions (Li et al., 2011; Guo et al., 2021). (Liao et al. (2021) utilized ultrasound to load BMSCs with mechanical stimulus, and found that the obtained EVs could inhibit inflammation *via* NF-κB pathway, and enhance cartilage matrix deposition (Figure 2). Apart from 3D culture and mechanical stimulation, hypoxia microenvironment regulates MSC performance *via* HIF-1α (Malladi et al., 2007). EVs derived from hypoxia pre-treatment MSCs were proved to promote repair capability of chondrocytes through miR-216a-5p/JAK2/STAT3 pathway (Rong et al., 2021).

### Modifying EVs directly for EVs engineering

Indirect methods pre-synthesize components to enrich EV cargos *via* donor cell modification. Direct engineering strategies, including enriching EV cargos and modifying EV membranes, can endow EVs with specific and controllable functions.

#### Enriching EVs cargos

Multiple strategies can be utilized to transfer cargos into EVs, including direct co-incubation, physical and chemical methods. Hydrophobic molecules can pass across EV phospholipid membrane, which makes them suitable for the direct mixture method (Fuhrmann et al., 2015). Li et al. (2021) co-incubated EVs with chitosan oligosaccharides (COS) for 1 h at 37°C to make COS go into EVs and construct COS-EVs. The result indicated that compared to EVs, COS-EVs exhibited better ability in promoting anabolic related genes expression of chondrocytes. Strategies like electroporation (Kooijmans et al., 2013; Pomatto et al., 2018), sonication (Lamichhane et al., 2016), saponin permeabilization (Pomatto et al., 2019), freeze-thawing (Luan et al., 2017), and CaCl_2_ mediated method (Zhang D. et al., 2016) can be utilized to directly load EVs with bioactive molecules. To enhance drug encapsulation rate, Xu et al. (Xu et al. (2021) encapsulated kartogenin (KGN) into EVs *via* electroporation, and the encapsulation rate was advanced to 40% compared to 8% of direct co-incubation. The enhanced KGN delivery efficiency could promote cartilage repair.

#### Modifying EVs membrane

To further improve the target and drug delivery efficiency of EVs, EVs membrane can be modified. Xu et al. (Xu et al. (2021) modified EV surface with E7 peptide *via* plasmids transfection of donor cells to target synovial fluid-derived MSCs for enhanced OA treatment. Through plasmids transfection, liang et al. (Liang et al., 2020) fused chondrocyte-affinity peptide (CAP) with EV surface protein, and improved the chondrocyte target ability of CAP-EVs. Apart from protein fusion, regulating EV surface charge potential can promote EV penetration since the block influence of negative cartilage matrix. Feng et al. (2021) utilized ε-polylysine-polyethylene-distearyl phosphatidylethanolamine (PPD) modifying EVs to construct positively charged MSC-EVs for better cartilage matrix penetration. Wei et al. (Wei et al.) endowed EVs with amphiphilic positive potential through surface modification of cationic 1,2-dioleoyl-3-trimethylammonium propane (DOTAP), and the results showed that DOTAP modified EVs could promote the penetration of EVs into cartilage matrix, extend EV retention and attenuate OA destruction.

## Conclusion and further perspectives

EVs derived from different MSCs, loaded with abundant cargos including miRNAs, lipids, and proteins, show great potential in OA treatment. The mechanisms underlying the MSC-EVs therapeutic effects lie in that MSC-EVs can play roles in modulating cell fate of chondrocyte, remodeling bioenergetic metabolism, regulating immune system and promoting cartilage matrix synthesis. To further enhance target and delivery efficiency of EVs, gene transfection manipulation, co-incubation and cell culture microenvironment engineering can be utilized to modify donor cells, and EV cargo enrichment and EV membrane modification can be applied to directly modify EVs.Wei Y et al., 2021.

Although the wide application of MSC-EVs, in OA treatment, there are still challenges requiring further explorations.

1) Current studies mostly concentrate on the phenotype of chondrocytes following EV treatment, but the molecular mechanisms underlying the phenomenon are still unclear. For precise medical treatment, the discovery of specific target is crucial. Understanding the molecular mechanism in OA development and treatment can provide a new approach for treatment and facilitate precise intervention for patients.
2) Apart from the therapeutic effect of MSC-EVs, EVs also play a role in the pathological process of OA development. Studying the pathogenic effect of EVs can help better understand the molecular mechanism of OA progress, and facilitate the exploration of new therapeutic strategies. Moreover, EVs can serve as biomarkers for early OA diagnosis, and more specific and effective EVs as biomarkers are required to be found.
3) The MSC-EVs treatment can attenuate OA progression, but the effect of MSC-EVs on reversing chondrocyte function is short of demonstration. Whether applying EVs in early or late period of OA can reverse OA pathological changes, but not only mitigate OA progression, which needs more explorations.
4) The kinetics and bio-distribution of EVs, and dosage of EVs used in *vivo* experiments and further in clinical trials is still unclear, which needs more researches to clarify.
5) The MSC therapy has undergone clinical trial period, and can be applied in clinic, but the safety of MSC-EVs for clinical application requires more studies to verify.
6) Indirect EV engineering strategies are costly, time-consuming and hard to manipulate; utilizing new gene-editing technologies, like CRISPR-Cas9 can make the gene manipulation more efficient and accurate. Direct EV modification might exert negative influence on morphology and size of engineered EVs, and efficient and scatheless EV engineering strategies are required.
